# Mindfulness meditation and psychedelics: potential synergies and commonalities

**DOI:** 10.1007/s43440-023-00551-8

**Published:** 2023-11-06

**Authors:** Paweł Holas, Justyna Kamińska

**Affiliations:** Faculty of Psychology, University of Warsaw, Warsaw, Poland

**Keywords:** Psychedelics, Mindfulness, Meditation, Mindfulness-based interventions, Predictive coding, Mystical experiences, Psilocybin, Decentering

## Abstract

There has been increasing scientific and clinical interest in studying psychedelic and meditation-based interventions in recent years, both in the context of improving mental health and as tools for understanding the mind. Several authors suggest neurophysiological and phenomenological parallels and overlaps between psychedelic and meditative states and suggest synergistic effects of both methods. Both psychedelic-assisted therapy and meditation training in the form of mindfulness-based interventions have been experimentally validated with moderate to large effects as alternative treatments for a variety of mental health problems, including depression, addictions, and anxiety disorders. Both demonstrated significant post-acute and long-term decreases in clinical symptoms and enhancements in well-being in healthy participants, in addition. Postulated shared salutogenic mechanisms, include, among others the ability to alter self-consciousness, present-moment awareness and antidepressant action via corresponding neuromodulatory effects. These shared mechanisms between mindfulness training and psychedelic intervention have led to scientists theorizing, and recently demonstrating, positive synergistic effects when both are used in combination. Research findings suggest that these two approaches can complement each other, enhancing the positive effects of both interventions. However, more theoretical accounts and methodologically sound research are needed before they can be extended into clinical practice. The current review aims to discuss the theoretical rationale of combining psychedelics with mindfulness training, including the predictive coding framework as well as research findings regarding synergies and commonalities between mindfulness training and psychedelic intervention. In addition, suggestions how to combine the two modalities are provided.

## Introduction

Mindfulness practice and psychedelic therapy have gained increasing attention in the last decade for their potential synergistic effects in promoting mental well-being and personal growth [1]. This overlap in desired outcomes suggests a potential complementarity, such as reducing the tendency for rumination via decreasing activation of the default mode network [2, 3] in the treatment of depression and anxiety disorders [4, 5].

Several studies [6, 7] and expert opinions [8] suggest that combining these two approaches may enhance therapeutic outcomes. Some experts argue that mindfulness meditation (MM) can complement psychedelics effectively, i.e., by helping individuals to develop a heightened sense of self-awareness and emotional regulation, which can be particularly useful during a psychedelic experience [9]. Experts also suggest that vice versa, psychedelics can create a favorable mental context for meditation, which is particularly important at the beginning of meditative practice marked by high obstructions to engage [8]. In simpler terms, psychedelics have a remarkable ability to temporarily lead individuals toward significant and often abrupt changes in their thinking patterns, values, attitudes, and behaviors, ultimately promoting more adaptive and positive qualities [10].

The combination of psychedelics and mindfulness can also be seen historically. During 1960s, when psychedelics gained popularity, they were often associated with practices like meditation and mysticism, reflecting an early recognition of their synergistic potential [11].

Although MM and psychedelics are distinct approaches, both share numerous parallel phenomenological, psychological, and neurobiological effects. The current review aims to provide a summary of these complimenting and synergistic mechanisms and to suggest methods for integrating both approaches to enhance their positive effects.

## Mindfulness meditation and mindfulness-based interventions

Mindfulness, rooted in Eastern contemplative traditions, has gained significant recognition in Western psychology and healthcare in the recent 2 decades. Mindfulness meditation usually involves directing one's attention to the breath, bodily sensations, thoughts, and emotions, promoting a sense of presence. One of the most popular definitions describes mindfulness as the awareness that emerges when deliberately and nonjudgmentally paying attention to the present moment experience [12]. Originally developed within Buddhist and other wisdom traditions to alleviate mental suffering and gain insights, mindfulness has been integrated into the Western mainstream of medicine under the umbrella term mindfulness-based interventions (MBIs). Among MBIs, there are two main mindfulness training: mindfulness-based stress reduction (MBSR) developed by the pioneer of mindfulness John Kabat-Zinn in 1978, and mindfulness-based cognitive therapy (MBCT) which has gained more empirical evidence of effectiveness in mental disorders. MBIs encompass structured programs that incorporate mindfulness practices within various contexts including clinical settings, education, and workplace wellness programs. Studies show that MBIs can enhance emotional regulation, attentional control, and self-awareness [13], inferring positive effects on well-being, alleviation of anxiety, depression, and stress symptoms [14]. The benefits of MM extend beyond psychological well-being, however. Mindfulness meditation has been associated with improved physical health outcomes and pain management [14].

Mindfulness-based interventions have also shown promise in behavior change and addiction treatment [15]. They help develop a greater awareness of addiction triggers and automatic reactions, enabling practitioners to respond more skillfully to challenging situations [15]. Beyond that, this broader awareness can lead to healthier choices and reduced impulsivity.

While mindfulness practices offer numerous advantages, their effective implementation requires appropriate training and guidance. Skilled instructors play a crucial role in guiding participants through the practices and addressing any challenges that may arise. Additionally, research highlights the importance of regular practice for sustained benefits [16]. These potential shortcomings of MM provide room for complementation from psychedelics.

Notably, in recent decades, the rapid advancement of information technology has led to a surge in the utilization of internet-based psychological interventions (IPIs) for addressing emotional challenges [17]. Internet-based Mindfulness-Based Interventions (iMBIs) have also been created, including iMBCT [18, 19]. Several meta-analyses of iMBI studies demonstrated that iMBIs, much like traditional MBI, effectively reduce stress and alleviate emotional distress, including depressive and anxiety symptoms [20, 21]. This evidence is further corroborated by a meta-analysis of iMBI interventions specifically aimed at individuals diagnosed with depression or anxiety disorders [22, 23]. Similarly to face-to-face MBIs, studies showed that iMBCT infers its positive effects on psychopathological symptoms through the increase in cognitive defusion and self-compassion abilities [24, 25]. Mindfulness meditation has become increasingly accessible through modern technology, specifically mindfulness apps. These apps offer guided meditation, breathing exercises, and tools to promote self-awareness and stress reduction. Research has shown that mindfulness apps not only have gained increasing popularity, but also can be efficient in improving psychological well-being, reducing symptoms of anxiety, depression, and emotional distress [26, 27]. Given the limited quantity of studies included in those meta-analyses, their methodological weaknesses, and the absence of randomized controlled trials, it is advisable to exercise caution when interpreting the effects of mindfulness apps. Future studies should also assess their utility and effectiveness in combination with psychedelic interventions.

## Psychedelics and psychedelic interventions

Psychedelics as a class of psychoactive substances, which induce intense and transformative experiences that might provide rapid insights, potentially accelerating some of the benefits derived from mindfulness practices [28]. Psychedelics themselves induce altered states of consciousness, characterized by profound perceptual, cognitive, and emotional experiences [3]. The term, however, is frequently utilized in a sweeping manner to encompass diverse categories of hallucinogens related to psychedelics. To prevent any potential numerical errors, the term "classic psychedelics'' is used, referring to the category of substances that bind to serotonin receptors in the brain, with 5HT2A receptor selectivity [29]. This group includes mescaline, Lysergic Acid Diethylamide (LSD), and naturally occurring alkaloids, such as 4-phosphoriloxy-N,N-dimethyltryptamine (psilocybin, present in hundreds of Psilocybe mushroom species), ayahuasca, and DMT, among others [30]. Additionally, there are other types of psychedelics, such as 2-(2-Chlorophenyl)-2-(methylamino) ketamine and 3,4-MethylenedioxyMethamphetamine (MDMA). Ketamine is a dissociative psychedelic, which acts as NMDAR (N-methyl-D-aspartate receptor) antagonists, effectively engaging the glutamatergic system [31]. MDMA is known as “empathogenic psychedelics”, or “entactogen”, acting primarily by increasing the release of serotonin, dopamine, and norepinephrine and inhibiting their reuptake [32]. As previously noted, mindfulness practices facilitate behavioral change by enabling individuals to identify triggers and habitual responses, whereas psychedelic therapy can amplify this awareness, resulting in even deeper behavioral transformations [33]. Furthermore, while the effects of psychedelics are not permanent, they can produce long-lasting changes from even a single session, potentially complementing the regular practice required by mindfulness [7].

The potential therapeutic applications of psychedelics, often referred to as psychedelic interventions or psychedelic-assisted therapy (PAT), have opened new avenues for addressing mental health conditions. Classic psychedelics, such as synthetic LSD and psilocybin, which is found in certain mushrooms, have shown promise as aids in psychotherapy e.g., for mood disorders and substance dependence [1, 5, 33]. Psychedelic-assisted psychotherapy involves guided sessions where individuals receive a controlled dose of a psychedelic under the supervision of trained therapists. This approach aims to enhance emotional processing, encourage introspection, and facilitate breakthroughs in therapy [34].

## Altered states of consciousness

Meditation and psychedelics have garnered significant attention in recent years, both in the context of altered states of consciousness and as tools for understanding the mind. Both psychedelic and meditation, are historically and contemporarily considered as methods of alternating states of consciousness. Consciousness, in general, includes a wide variety of experiences, ranging from simple perceptual awareness to introspective thought [35]. One may argue it creates our inner "self", which is at the center of what shapes our uniqueness [36]. Although this assumption might suggest that our self of identity is permanent and solid, such a claim is questioned by altered states of consciousness in which the disruption of “self” are observed [36, [37], such as in conditions like temporal lobe epilepsy auras [38] or spiritual or mystical experiences [39]. The latter can be induced by various means, including but not limited to [40] psychedelics or meditation [36, 37].

Such phenomena, often termed "self-loss" or "ego dissolution", manifest as a blurring or complete disappearance of the sense of self, promoting a sense of oneness with the world or surroundings [41]. Consequently, in this state, individuals may more readily engage with their cognitive beliefs and emotional states with enhanced detachment and decentration, potentially leading to therapeutic outcomes [42, 43]. One of the explanations for these salutogenic effects is that the 'ego-dissolution' phenomenon is related to the deactivation of the groups of interconnected brain regions called Default Mode Networks (DMN) [2]. This region includes the following regions: medial prefrontal cortex (MPFC), anterior cingulate cortex and posterior cingulate cortex (PCC), cuneus/precuneus, and temporoparietal junction/angular gyrus (some also list hippocampus, parahippocampal gyri, and frontopolar cortex as regions “loosely integrated”) [44]. DMN serves as the neurophysiological basis for the 'self' or 'ego' because of its activation when individuals engage in self-referential thought [45]. It has been demonstrated with functional magnetic resonance imaging (fMRI) that during psychedelic experience, the DMN is modulated, which sharply reduces blood flow and connection within its nodes [46], leading to decreased functional connectivity within the DMN and increased between-network connectivity [47, 48]. These alterations are believed to foster a cognitive landscape that's more malleable, less restrained by traditional boundaries, and exhibits decreased ego-centered thinking [49, 50]. Similarly, mindfulness meditation has been associated with decreased activity in the DMN during periods of focused attention [2]. Moreover, long-term meditators display reduced DMN activity even during resting states, suggesting a lasting effect of meditation on self-referential thought and DMN connectivity [51].

## Mystical experiences in mindfulness training and psychedelics

Mystical experiences, often referred to as transcendent encounters or spiritual revelations, are deeply profound moments that transcend ordinary consciousness. These experiences are reported across cultures, religions, and historical periods, often accompanied by a sense of unity, awe, and a connection to the divine. They often involve altered states of consciousness and are described as moments of heightened awareness that defy ordinary sensory perception. While these experiences are deeply personal and subjective, they often result in a profound transformation in the individual's perspective and worldview [4]. Mystical experiences can be triggered by various means, including meditation, prayer, and religious rituals or even spontaneous occurrences. These experiences, however, most often are induced by mindfulness meditation and the use of psychedelics. While both methods share certain commonalities in producing mystical encounters, they also exhibit notable differences.

Studies [52] suggest that MM and psychedelics share a history of consciousness alteration, although mindfulness often evolves through gradual, focused practice, while psychedelics induce altered states more rapidly. Moreover, psychedelic-induced experiences are often characterized by their vivid and elaborate sensory content, a quality that is typically lacking in meditative states. Psychedelics can evoke intense sensory perceptions, including visual hallucinations, enhanced color perception, and heightened auditory sensations. This phenomenon is in stark contrast to meditative states, which tend to involve a reduction in sensory awareness and a shift toward inner contemplation and mental stillness. Meditation often seeks to transcend sensory input and achieve a state of heightened awareness devoid of external distractions.

Research [53] indicates that classic hallucinogens can evoke profound states of unity, described as experiences of interconnectedness and oneness with all phenomena, akin to deep meditative states. Such states of unity involve ego dissolution, which refers to a fading or elimination of the individual's normal sense of self, blurring the habitual boundaries between self and other and fostering an encompassing feeling of wholeness and integration with the surrounding environment [67]. However, psychedelics' effects are largely dose-dependent and can vary among individuals, leading to potentially unpredictable outcomes. Mindfulness training, on the other hand, offers a controlled and self-guided approach to achieving similar states of consciousness.

Studies showed that similarly to MM, acute effects of psychedelics include non-dual experiences [54] and elevated interpersonal connectedness [55]. Moreover, psychedelics have been found to increase trait mindfulness, and the skills of nonjudgement, compassion and non-reactivity [56, 57].

Differences emerge in the mechanisms and sustainability of these experiences. Mindfulness relies on self-regulation and introspection, gradually cultivating altered states through dedicated practice. Psychedelics, on the other hand, offer a shortcut to such states, but their effects can be temporary and influenced by external factors [29, [58].

However, the challenge of maintaining a mindfulness practice over an extended period can often lead to decreased motivation and high dropout rates [59]. Psychedelics, by temporarily inducing peak experiences and afterglow states that resemble the deep meditative states, may serve as a motivating factor for individuals to sustain their mindfulness practice over time. Research showed that sustaining mindfulness practice is essential for achieving and maintaining health benefits and that mindfulness training effects are dose-dependent, at least for the first 500 h [60].

## Neurobiology of psychedelics and mindfulness meditation

Classic psychedelics like LSD, psilocybin, mescaline, and DMT primarily act as agonists to the serotonin 5-HT2A receptor, and considering the extensive range targeted by the serotonergic system, they have a significant effect on the brain's neurochemistry [61]. The high presence of the 5-HT2A receptor in regions like the prefrontal cortex—integral for higher-order cognitive functions—underscores the transformative nature of these compounds on cognition and perception [62, 63]. The other explanation of psychedelics' ability to facilitate altered states of consciousness characterized by heightened introspection and diminished ego boundaries is their influence on 5-HT1A [64] and 5-HT2B [65], and interacting with the dopaminergic [66] and glutamatergic systems [67]. Interestingly, the salience network, which determines which stimuli warrant our focus, has also been investigated as a neural correlate of self [68, 69]. Research indicates that psychedelics disrupt the functioning of the salience network, potentially leading to altered perceptions of self and world [70]. Such disruption is believed to be associated with the experience of ego dissolution [71]. Complementing this, there's also a notable link between psychedelics and the induction of profound, mystical experiences (which are usually marked by ego dissolution), which are said to be characterized by heightened brain entropy [50]. Brain entropy in the realm of neuroscience serves as an indicator of a system's uncertainty. The higher entropy means more complex, diverse, and unpredictable neural activity [50]. In the context of the brain, this denotes a blend of disorganization paving the way for a more diverse range of dynamic neural states [72].

Meditation, particularly mindfulness practices, has garnered significant attention for its profound neurobiological effects as well. Similarly to the mechanism of default mode network DMN influenced by psychedelics, mindfulness meditation decreases DMN activity which leads to changes in the precision-weighting of beliefs and attention [41]. MM was also found to reduce the activity of the midline prefrontal cortex and posterior cingulate cortex, which are integral parts of the DMN, suggesting that mindfulness training can alter self-awareness pathways and promote present-moment consciousness [73]. Beyond these neural circuits, MM encourages widespread neuroplastic changes. For example, comparative studies between meditators and non-meditators have revealed differences in brain connectivity, activation patterns, and both gray and white matter density [74]. In addition, the thalamus, a key region in sensory gating, is also involved in the meditative states [36, 41].

Acknowledging the aforementioned, MM and psychedelics both play roles in dissolving self-awareness, fostering insight, and promoting personal growth and well-being. Studies have identified overlapping brain connectivity patterns in meditative and psychedelic states, especially within resting-state networks, implying therapeutic properties. [2, 4, 6, [75].

In the next sections, we will outline the potential commonalities, synergies and differences of Mindfulness Training and Psychedelic Interventions. The following list is not intended to be exhaustive, but it is meant to indicate possible ways through which mindfulness training can support psychedelic interventions, as well as ways in which psychedelics can aid meditation practice.

## Effects of mindfulness training and psychedelic intervention on prosociality

Mindfulness training and psychedelics have shown potential effects on prosociality. While research is still emerging, some studies suggest that both approaches may enhance empathy, compassion, and social connectedness [55, [76, 77] as well as increased self-compassion and prosocial attitudes [78, 79]. Psychedelic intervention, especially in combination with mindfulness practices, has been proposed to facilitate positive changes in social attitudes and behaviors due to its impact on self-awareness and perspective shifts [80]. Despite these promising findings, it's crucial to note that the effects of psychedelic therapies on prosociality are complex and context-dependent.

## Effects of mindfulness training and psychedelic intervention on decentering

Decentering—defined as the capacity to objectively observe one's immediate experience, altering its inherent nature—is a pivotal mechanism underpinning mindfulness meditation, namely mindfulness-based cognitive therapy (MBCT) [81]. In general, both Mindfulness-Based Interventions and Psychedelic Interventions have been recognized for their potential to cultivate decentering [82]. Non-ordinary states of consciousness induced by practices like mindfulness meditation and psychedelics have shown effects on different cognitive processes, including decentering and similar phenomena of cognitive defusion [91]. In addition, studies showed that ceremonial use of psychedelics (ayahuasca) is related to an increase in cognitive reappraisal which helps to look at and experience adverse life events with distance [83].

## The effects of mindfulness training and psychedelic intervention on emotion regulation

Mindfulness training has shown promising impacts on emotion regulation [84, 85]. Studies suggest that MBIs can cause increased activation in the dorsolateral prefrontal cortex, a region associated with cognitive control of emotions, indicating improved emotion regulation [86]. While research on psychedelics' effects on emotion regulation is limited, it indicates that psychedelic experiences influence emotional processing and self-awareness [87, 88]. Psilocybin's effects might lead to a reevaluation of emotional experiences and enhanced emotional flexibility. Recent findings suggest that both approaches enhance affective self-regulation by influencing emotional reactivity and emotion regulation [89], however, their synergy in this respect warrants further research.

## The effects of mindfulness training and psychedelic intervention on psychological flexibility

Mindfulness practices encourage individuals to respond to all kinds of experiences, whether positive or negative, without judgment and with openness which fosters psychological flexibility [90]. This acceptance aligns with psychological flexibility's core components, enabling individuals to act by their values even in the presence of challenging emotions [79, [91]. Psychedelics, on the other hand, can lead to profound insights into personal values, and in this way enhance psychological flexibility [92].

Both methods encourage individuals to embrace uncertainty and change, a fundamental aspect of psychological flexibility. Psychological flexibility involves moving beyond limitations imposed by thoughts and emotions. Mindfulness training teaches individuals to observe their thoughts without attachment, reducing cognitive rigidity. Psychedelics often induce experiences that challenge pre-existing beliefs, allowing individuals to transcend the constraining influence of self-concepts and through this way promote adaptability and open-mindedness [3, 38]. Both offer avenues to increased psychological flexibility by fostering acceptance, values alignment, embracing uncertainty, and challenging ego boundaries. Integrating mindfulness skills and psychedelic insights holds promise for sustained psychological flexibility by facilitating a balanced response to internal and external stimuli, and adaptive responses to life's challenges [93].

## The effects of mindfulness training and psychedelic intervention on psychopathology reduction

Both methods have shown potential for reducing psychopathology and both, psychedelic-assisted therapy with psilocybin and MBIs, in particular MBCT have preliminary evidence for effectiveness in chronic, treatment-resistant depression [94]. MBIs have demonstrated their efficacy in reducing psychopathological symptoms [14]. Psychedelic intervention, particularly with substances like psilocybin and ayahuasca, has also shown promise in complementing mindfulness practices to improve mental health outcomes [98]. Precise mechanisms underlying the psychopathology reduction are complex and multifaceted, involving changes in neural networks, psychological processes, and self-reported altered resting states [95]. The synergy between mindfulness and psychedelics has the potential to create a holistic approach to psychopathology reduction. While mindfulness encourages self-awareness and emotional regulation, psychedelics can promote transformative experiences that lead to insights and shifts in perspective [8]. Both methods have shown potential for reducing psychopathology through a variety of mechanisms, including those described in previous sections: decentering ability enhancement, increasing psychological flexibility and increasing emotion regulation. Additional mechanisms include but are not limited to:

Avoidance reduction: Both MBCT and psychedelic intervention have been associated with a reduction in avoidance behaviors [101].

Synergic effect on reduction of egocentricity. A combination of MM and PAT increases empathy and reduces egocentricity, potentially aiding psychopathology reduction [7]. Moreover, this effect has been shown even for a single dose of psilocybin [7]. The process of decoupling within self-referential networks combined with the alteration in one's sense of self induced by psilocybin during a meditation retreat, can serve as a predictive factor for lasting positive changes in psycho-social well-being [7]. This suggests that the experience of self-dissolution when integrated with mindfulness practices, may contribute to sustained improvements in psychological and social functioning.

Neural plasticity and rewiring: Both approaches have shown the potential to induce neuroplastic changes in the brain. Psilocybin may temporarily enhance neuroplasticity through its influence on glutaminergic processes from the bottom up [96], and foster neural rewiring by promoting new synaptic connections, potentially breaking rigid thought patterns associated with psychopathology [97]. On the contrary, mindfulness meditation can increase gray matter volume in regions associated with emotion regulation and self-awareness and predominantly relies on top-down regulatory mechanisms and gradually fosters plasticity as training progresses [73]. These two approaches could complement each other, with MM potentially extending the neuroplastic state triggered by psilocybin, and psilocybin potentially accelerating the rise of brain-derived neurotrophic factor (BDNF) during MM training [see also 4]. This synergy may lead to enhanced neural plasticity and cognitive benefits.

Neuroendocrine and neuroimmunological factors: The weakening of stress responses has been indicated as a core mechanism through which MBIs exert their beneficial effects on both mental and physical health [14]. MBIs decrease subjective psychological stress levels [18, [98] and helps people handle stress more effectively by making them less reactive to it and by improving the brain's ability to regulate stress-related processes, through its effect on the hypothalamic–pituitary–adrenal (HPA) axis, cortisol secretion [107, [99], and additionally reduction of pro-inflammatory cytokines, including IL-6 [107, 109]. The neuroendocrine and neuroimmune systems are interconnected via hormone and cytokine signaling [100], and MM and psilocybin act with different mechanisms and possibly complementarily on these systems. While MBIs enhance HPA axis regulation leading to a decrease in cortisol level, psychedelics appear to mitigate inflammation through 5-HT2A agonism, leading to inhibiting the production of IL-6 and consequently reducing the stimulation of the HPA axis [4]. Notably, psilocybin is associated with a pronounced increase in cortisol levels [101]. This surge may be pivotal in facilitating the 'unlearning' of negative associations, prioritizing the formation of new memories over the retrieval of older ones [102]. The presence of 5-HT2A receptors in the prefrontal cortex has been correlated with stress response pathways [103] and, as such, a progressive down-regulation of these receptors due to psychedelics is thought to be essential for alleviating stress [104]. In the realm of neuroimmunity, psilocybin is postulated to alter cellular signaling within the immune system, given the abundant presence of 5-HT2A receptors in immune cells [105]. Psilocybin modulates the immune response by suppressing inflammatory pathways through actions mediated by this 5-HT2A receptor [106], 117]. Preliminary research also suggests that certain psychedelics could curtail the production of the pro-inflammatory cytokine IL-6 [107], which might elucidate the enduring antidepressant effects observed with psilocybin [116].

## Studies utilizing a combined methodology

A study done by Griffiths et al. [6] showed that a combination of psychedelics and meditation generates long-term improvements in psychological functioning as well as trait measures of prosocial attitudes and behaviors [6]. In this study, groups of healthy participants were randomized into three groups, (1) low-dose psilocybin with standard support for spiritual practice (SS); (2) high-dose psilocybin with SS, and (3) high-dose psilocybin with High Support (HS) for spiritual practice. Importantly, the number of guide-participant meetings varied between the standard and high-support conditions. Between the study's acceptance and the 6-month follow-up, participants in the SS groups had a combined contact time with their guides that amounted to approximately 7 h and 20 min, whereas the HS group accumulated a total of 35 h of contact between the guides and the participants from the acceptance of the study to the 6-month follow-up. Psilocybin was administered 1 and 2 months following spiritual (mainly meditative) practice. The results showed, that high-dose psilocybin combined with HS spiritual practices led to more immediate and long-lasting effects at a wide range of measures including interpersonal closeness, gratitude, and life meaning/purpose [6]. Importantly, determinants of enduring effects were psilocybin-occasioned mystical-type experience and rates of engagement with meditation practices.

Smigielski and colleagues [7, 108] reported similar outcomes following the administration of psilocybin to individuals with long-term experience of mindfulness meditation during a 5-day Zen meditation retreat. This was a double-blind, placebo-controlled trial involving 38 participants who were screened with psychometric instruments and in addition with functional magnetic resonance imaging during the resting state and two meditation forms, pre to post-intervention. The results of brain imaging showed that psilocybin, particularly affected regions associated with self-referential processing (DMN). Notably, changes in this network correlated with participants' subjective experience of ego dissolution during the psilocybin-assisted mindfulness session [118]. Four months of follow-up indicated that psilocybin produced bigger favorable gains in psychosocial functioning than placebo [7, 118]. Intriguingly, the results suggest that meditation appears to enhance psilocybin's positive effects while mitigating potential negative reactions.

Altogether, findings from the aforementioned studies suggest that mindfulness meditation when combined with psilocybin administration has the potential to alter both how we think and the content of our thoughts, making them more positive and open-minded. This combination may also help individuals maintain control over strong emotions that can arise when using psilocybin and could lead to improved communication skills. Ultimately, these effects could improve various psychological variables, including mood, cognitive control, and satisfaction in relationships.

## Mindfulness training and psychedelic intervention—predictive coding framework

Predictive coding is a new emerging framework that provides a promising avenue for understanding the effects of both meditation and psychedelics on consciousness. The predictive processing framework has been suggested as a unifying theory of the embodied brain and its cognitive functions [109]. The main assumption of this neuroscientific theory is that the brain operates as a prediction machine that constantly generates predictions about the world based on prior knowledge and sensory input. When predictions match sensory data, there is minimal prediction error, leading to a feeling of coherence and confirmation. However, when there is a mismatch, the brain updates its predictions, resulting in a sensation of surprise. An essential component for not being stuck in the pathological forms of rigidity in thinking that underlie a variety of psychiatric problems, including depression and addictions [110], is to be able to effectively manage predictive errors based on sensitivity to error dynamics (the change in the rate of error reduction, [111]). Rate fluctuations signify that error reduction has deviated from expected outcomes, either improving or worsening. When agents perform beyond or below expectations, their bodies react with positively or negatively valenced emotional responses. In other words, positively and negatively valenced affective states are indicators of better than or worse than expected error reduction, respectively. Agents who harness these emotional states to govern their actions are motivated to consistently enhance error reduction, and to function optimally, it is crucial to be attuned to opportunities for making progress in error reduction [112].

In the context of meditation, practitioners often aim to reduce prediction errors by focusing on the present moment, thus decreasing mind-wandering and promoting a sense of calm and clarity. The mechanism behind this involves minimizing the brain's forecasting of future events or recall of past ones, and as prediction errors reduce, the mind naturally wanders less since it is not engaging in creating alternative scenarios or reflections. Consequently, this reduction in speculative and divergent thinking, which is achieved through sustained, non-judgmental awareness of the present, helps not only to stabilize mind, but also fosters mental calmness and stillness, as the cognitive load lessens and the mind is not juggling between various temporal thoughts. Meditation practices like mindfulness meditation train individuals to be aware of their sensations, thoughts, and emotions without judgment, allowing them to fine-tune their predictive mechanisms. Along this process, over time, being in the here and now decreases predictive processing itself, leading to the gradual reduction of counterfactual abstract processing, including predicting self [113]. However, meditation can also induce extraordinary, novel mental experiences that cannot easily be accounted for by existing systems of beliefs (models), which leads to an increase of their uncertainty and requires revision of them. Importantly, meditation by its emphasis on body and mind stillness, may promote facts free learning [114] that is sudden insights, discovery or perspective without new information.

Psychedelics, on the other hand, can be seen as agents that temporarily disrupt the usual predictive processes. They induce alterations in perception, cognition, and self-awareness by introducing unpredictability into the brain's operations, thus enabling new learning. This disruption can lead to profound and often mystical experiences, and in the case of psychopathology such as depression where negative beliefs form a maladaptive generative model of the world, these transformative experiences may lead to positive therapeutic outcomes. Specifically, *relaxed beliefs under psychedelics* (the REBUS model) [115] states that psychedelics serve to relax prior beliefs. In this context, it refers to softening the rigid and often impermeable cognitive and perceptual frameworks or assumptions that govern our experiences and interpretations of the world. When a collection of these prior beliefs forms a maladaptive generative model of the world, individuals may become “stuck” in a depressive episode. Through altered experiences, psychedelics therefore allow patients to shift from a maladaptive model to a healthier one, as these top-down depressive beliefs have less influence on the generation of conscious experience. Psychedelics might temporarily reduce the precision of predictions, allowing individuals to perceive the world with fresh eyes, potentially leading to therapeutic insights [116]. In sum, meditation aims to refine predictive processes for greater clarity, whereas psychedelics temporarily disrupt them, opening the door to novel perceptions and insights.

## Conclusions

The relationship between mindfulness practice and psychedelic intervention appears to hold promise as a synergic match. Research and historical contexts suggest that these two approaches can complement each other, potentially leading to more profound therapeutic experiences, enhancement of the positive effects and better mental health outcomes. Mindfulness training enhances the experience of ego dissolution induced by psychedelics, while these compounds can deepen meditation practices and engagement in spiritual practices, in both expert and novice meditators. Additionally, when psychedelics are administered in natural settings, they spontaneously boost mindfulness capabilities, which can potentially support and enhance contemplative practices.

Those who want to achieve synergistic and improved results from a combination of psychedelics and mindfulness meditation may benefit from abiding by some basic rules:

1. *Professional Guidance* Ensure that any combination of these interventions is conducted under the supervision of trained professionals. Seek guidance from therapists or experts experienced in both psychedelic therapy and mindfulness practices.

2. *Integration* After a psychedelic experience, integrating the insights gained during the journey into mindfulness practice can be highly beneficial. Meditation and mindfulness can help individuals process and apply the lessons learned from the psychedelic experience to their daily lives.

3. *Set and Setting* Pay careful attention to the environment and mindset in which you engage in these practices. Create a safe and conducive setting for both mindfulness and psychedelic experiences to maximize their potential benefits.

4. *Mindful Preparation* Incorporate mindfulness into your preparation for a psychedelic journey. Mindfulness techniques can help reduce anxiety and set a positive intention for the experience.

5. *Mindful Presence* During a psychedelic experience, practice mindfulness by staying present and non-judgmental. This can enhance the depth of the experience and facilitate self-awareness.

6. *Post-Session Reflection* After a psychedelic session, engage in mindfulness-based reflection to process emotions, thoughts, and insights gained during the experience.

7. *Consistency* Maintain a regular mindfulness practice to support ongoing mental well-being and emotional resilience. Combining mindfulness with psychedelics can enhance the sustainability of positive changes.

8. *Research and Educatio*n Continuously educate yourself about both psychedelics and mindfulness. Stay informed about the latest research and developments in these fields.

9. *Personalization* Understand that the combination of these interventions may affect individuals differently. Tailor your approach to what works best for your unique needs and circumstances.

10. *Legal and Ethical Considerations* Adhere to legal and ethical guidelines regarding the use of psychedelics in your location. Ensure that any practices involving psychedelics are conducted responsibly and in compliance with applicable laws and regulations.

Above suggestions apply to the combination of psychedelic-assisted therapy and standard forms of low intensity MM. Future research should also consider evaluating if the combination of psychedelics and more intense mindfulness training in the forms of meditative retreats, could yield more significant benefits and, more specifically, for whom. Future studies may also benefit from evaluating the combination of specific types of mindfulness meditation with particular psychedelics to enhance specific abilities or alleviate particular forms of psychological distress. For instance, one unconventional and understudied approach involves combining Metta meditation, also known as loving-kindness meditation, with MDMA. Metta meditation is centered on nurturing feelings of love and compassion for oneself and others, while MDMA is a psychoactive substance renowned for its empathogenic effects. There is some evidence that MDMA, when administered in a therapeutic context, can enhance feelings of empathy and connection, which aligns with the goals of Metta meditation. Some observational studies have suggested that MDMA may enhance emotional empathy and self-compassion [117], the effects that are observed followed compassion-based meditation interventions [118].

While the review findings and experts' opinions highlight the potential synergy and some commonalities in their mechanisms of action, it's important to note that this area of research is still evolving, individual experiences may vary, and not everyone may benefit equally from the combination of mindfulness and psychedelics. Research on the potential synergistic effects between mindfulness training and psychedelics suffers from the presence of methodological limitations. Both fields of psychedelics and meditation are marked by strong bias effects [119, 120], so reported in studies beneficial effects can be overestimated. For example, the uncritical promotion of psychedelics as a strong medicine directly affects participant expectancy in ongoing psychedelic trials [121]. To establish a conclusive and robust understanding of any synergistic relationship between mindfulness training and psychedelics, future research must address these limitations. This includes conducting studies with larger sample sizes and implementing more rigorously controlled methodologies, including independent raters and active placebos. Replication studies with these improvements are essential to provide a clearer and more reliable picture of the potential benefits of combining mindfulness and psychedelics in therapeutic contexts. Further research, clinical trials, and careful guidance are necessary to fully understand the mechanisms and potential risks and benefits of combined treatment with psychedelics and mindfulness training. The current state of research, however, suggests that this "marriage" could indeed be fruitful and long-lasting.

## Data Availability

There are no data.
